# “ … I felt completely stranded”: liminality and recognition of personhood in the experiences of suicidal women admitted to psychiatric hospital

**DOI:** 10.1080/17482631.2020.1731995

**Published:** 2020-02-27

**Authors:** Julia Hagen, Birthe Loa Knizek, Heidi Hjelmeland

**Affiliations:** Department of Mental Health, Norwegian University of Science and Technology, Trondheim, Norway

**Keywords:** Care, liminality, mental health care, psychiatry, qualitative research, suicide

## Abstract

**Purpose**: The purpose of this study is to explore how patients experience their suicidality and how they experience being in a psychiatric hospital.

**Methods**: This is part of a field study, and the article is based on data collected in interactions with 11 women who were admitted to a psychiatric hospital and were struggling with suicidality. Data were collected through interviews, conversations, and participatory observation. We analysed the data by means of Systematic Text Condensation, followed by a deductive process where the data and preliminary findings were interpreted in light of the theory of liminality.

**Results**: We found that the patients’ experiences of suicidality and being a patient in a psychiatric acute ward involve “Liminality and weakened sense of personhood,” and from their perspective, “Recognition of personhood” is an important aspect of care.

**Conclusion**: Our study indicates that suicidality and psychiatric hospitalization involve liminality and weakened sense of personhood, aspects that are important to consider in the care of the patients. Professionals need to acknowledge more the importance of recognition of personhood in care, since this can strengthen the patient’s self-worth and empower the person. Lack of recognition may increase the patient’s suffering and suicidality.

## Introduction

Providing treatment and care of psychiatric patients experiencing suicidality can be challenging for professionals, and preventing suicide and suicidal acts among patients is difficult. Increased efforts to prevent suicide in mental health services during the last 10–15 years in Norway, among others through the *National guidelines for the prevention of suicide in mental health care* (Norwegian Directorate of Health and Social Affairs, 2008), does not seem to have decreased the suicide rate (Norwegian Institute of Public Health, 2019). Despite increased emphasis on evidence-based treatment and suicide prevention campaigns, suicide rates persist (White & Morris, 2019). In Norway, it was found that in the period 2010–2015, about 45% of all the persons who took their own lives had been in contact with specialized mental health- and substance misuse services during the last year (Walby, Myhre, & Kildahl, 2018). Nearly 90% of them had been in contact with specialized mental health services, and about 10% took their own lives while being under inpatient care (Walby et al., 2018). It is not clear if/how the persons’ suicides relate to their contact with these services or the treatment and care they received, but suicide prevention continues to be a big challenge in mental health services.

In psychiatric wards, the understanding of suicidality and approach to suicidal patients appears to be strongly influenced by biomedical and psychological perspectives, particularly the former (Hagen, 2018). A biomedical perspective on suicidality involves an emphasis on identification and treatment of mental disorders, which means that professionals try to decrease suicidality and prevent suicide among patients largely by diagnosing and treating a presumed underlying disorder. Thus, suicidality is related to individual psychopathology (Marsh, 2010, 2016), and approaches to prevent suicide are grounded in an expert, biomedical framework (White & Morris, 2019). A biomedical perspective in suicide prevention is reductionistic; other understandings of suicidality are not recognized, and other important aspects related to the suicidal person’s life and context are not considered (Hjelmeland & Knizek, 2017; White & Morris, 2019).

People who have been admitted to a psychiatric hospital because of suicidality have reported both positive and negative experiences (Berg, Rørtveit & Aase, 2017; Hagen, Knizek, & Hjelmeland, 2018). Good care involves connecting with empathic professionals who spend time with the patients, who confirm them and respond to their suffering and needs, whereas insufficient care implies that the needs for trusting connections, acknowledgement and support are unmet (Berg et al., 2017; Berglund, Åström, & Lindgren, 2016; Hagen et al., 2018; Talseth, Jacobsson, & Norberg, 2001; Talseth, Lindseth, Jacobsson, & Norberg, 1999; Vatne & Nåden, 2018). From the perspectives of patients at risk of suicide, recovery involves reconnecting with oneself and others, exploring how life can become worth living, and experiencing the ability to express oneself and to manage one’s own life (Sellin, Asp, Wallsten, & Wiklund-Gustin, 2017). In most qualitative studies (Berg et al., 2017; Hagen et al., 2018; Sellin et al., 2017; Talseth et al., 1999, 2001), the researchers explored patients’ experiences through one individual interview. We need to gain a deeper understanding of the patients’ situation and suicidality by collecting more comprehensive data.

By conducting a field study in a psychiatric acute ward, the aim of this study is to explore how patients experience their suicidality and how they experience being in a psychiatric hospital. Our research questions are: How do patients experience their suicidality and position as patients when they are hospitalized in a psychiatric acute ward, and how do they experience meetings with the professionals and the care they receive?

### Liminality—a relevant concept for mental health care

The concept “liminality” originates from van Gennep’s model of rites de passage (Van Gennep, 1960/2004). According to van Gennep’s model, all rites de passage or “transitions” involve three phases; separation from ordinary life, liminal period, and aggregation—integrating into everyday life again (Turner, 1977/1991; Van Gennep, 1960/2004). Turner studied the liminal period further and described it as a “betwixt and between” situation where the individual is in a transitional phase, which may involve a personal crisis, uncertainty, and change of status (Thomassen, 2009; Turner, 1967). Liminality applies to both space and time and may refer to different thresholds, specific places, or areas at specific moments or longer periods of time (Thomassen, 2009). Further, liminality applies to both individuals and larger groups/societies and may involve experiences such as sudden events affecting one’s life, critical life-stages or being part of a minority group (Thomassen, 2009). The concept liminality has been mostly used in anthropology, but it has also been used to shed light on different experiences, such as life-threatening illness (Bruce et al., 2014), and being a family caregiver to individuals with illness (Gibbons, Ross, & Bevans, 2014). Furthermore, the concept has been used to understand diagnostic categories such as “schizophrenic” (Barrett, 1998). Liminality also has been found in the experiences of hospitalized patients with dementia (Digby, Lee, & Williams, 2017), and in young men who have experienced emigration, substance abuse, and suicidal behaviour (Biong & Ravndal, 2009).

## Method

This is part of a field study and we employed qualitative methodology in the collection and analysis of data. By using qualitative methods, we develop knowledge based on exploring and interpreting the participants’ life-world, which contributes to a deeper understanding of what it feels like to be suicidal and hospitalized in a psychiatric acute ward. The data collected in interactions with 11 patients are the basis of this study.

### Research context

We conducted a field study in a psychiatric acute section in Norway. The first author (JH), who has a background as a mental health nurse and researcher in this field, collected data during a 3-month period in 2018, where she spent approximately 160 h (daytime) in the psychiatric acute section. The acute section was divided into three wards on the same floor; one intensive care ward/seclusion ward (10 beds) for patients needing seclusion, and two general acute wards (with 15 beds each) for patients admitted because of various mental health problems. Each ward had two living room areas; one located in the corner of the hallway, and one in a room with many windows. The section had two outdoor areas and one cantina available for the patients. The section was locked, so nobody could leave the building without the permission and assistance from the nurses. We got permission to conduct the study in the two general acute wards, and not in the intensive care ward due to safety reasons (e.g., aggression among patients).

The professionals consisted of therapists (physicians, psychiatrists, psychologists, specialist psychologists) and staff members working shifts in the wards (mainly nurses and mental health nurses; hereafter referred to as nurses). The therapists and the nurses had to take turns working in all three wards, shifting every 6 weeks. However, some of the nurses had to work in different wards during a 6-week period, depending on the situation in the acute section (e.g., sick leave among nurses, aggression, or other challenging behaviours among patients). The patients had 1–2 primary therapists (physician/intern, psychiatrist, psychologist), but no primary nurses (where1–2 nurses had the primary responsibility for the patient during their stay), although being assigned to one nurse each day and evening shift. However, the patients had to relate to another group of therapists and nurses when they shifted to another ward after 6 weeks. Thus, most patients had to relate to many professionals during their stay in the psychiatric acute section.

### Participants

The participants were 11 women (aged 20–41 years, median age 33 years) who were voluntarily admitted (some after persuasion) to a psychiatric acute ward and were struggling with suicidality. Three participants had harmed themselves or attempted suicide before they were admitted, whereas eight of them had serious thoughts of taking their own lives. Five participants were admitted to a psychiatric hospital for the first time, two had been admitted 1–3 times before, whereas four had been hospitalized more than 10 times over several years. Ten participants received treatment and care in an outpatient clinic and/or in the community health services. Their stay in the psychiatric acute ward lasted from 1 to 3 days (two participants), approximately 1 week (four participants), to several weeks (five participants). Two participants were transferred to a district psychiatric centre but returned to the acute ward shortly after. The three participants who had the longest stay in the acute ward were still hospitalized when JH completed the data collection.

JH recruited the participants by approaching the therapists and nurses first, asking whether there were any patients experiencing suicidality. Then, she asked the patient’s therapist whether it was appropriate to inform the patient about the study at that moment, and eventually (after some hours or 1 day) she asked the patient to participate. Sometimes, the therapist gave information about the study to the patient, and JH could approach them and ask them to participate afterwards.

### Data collection

The first author collected data through individual semi-structured interviews and conversations with 11 women who experienced suicidality. In addition, some data were collected through participatory observation in the ward. The semi-structured interviews were conducted in different meeting rooms in the acute section, and she had an interview schedule to help guide the conversation if necessary. The main questions were: Can you please tell me about your situation and the reason you came to the acute ward? How do you experience the meetings with the therapists and nurses? Can you please give examples where you did/did not feel well-taken care of by the therapist/nurse? What is most important for you to get better, including less suicidal thoughts? When appropriate, and in keeping with Kvale and Brinkmann (2009), JH probed for further elaboration (e.g., how did you feel? Can you please tell me more about that?) to obtain specific and in-depth information about the patients’ experiences. The interviews lasted from 30 to 85 min(median time 40 min), all interviews except one were audiotaped (approx. 8.5 h in total) and transcribed verbatim. JH wrote detailed notes during the interview that was not audiotaped, which in addition to other field notes were used as data. JH had one to four conversations with nine of the 11 women in addition to the interview. Some of the conversations occurred in the living rooms in the acute section, but most conversations were in the patients’ own room, where the patients felt more comfortable talking about themselves. As a participating observer, JH was present (usually silently participating) in one or two of the conversations these nine patients had with their therapist(s) (physician/intern, psychiatrist, psychologist) and nurse. She wrote field notes continuously (usually in the nurses’ office), but she wrote the most extensive reflections at the end of each day shift. The field notes contained specific information from the conversations with patients and observations in the ward as well as reflections about these conversations and observations.

### Analysis

We analysed the data by using inductive principles from Systematic Text Condensation, which is a form of thematic analysis searching for patterns within and across the material (steps 1–5; see the following) (Malterud, 2012, 2017). In addition, we read the data through theory, plugging one text (data) into another (theory) as described by Jackson and Mazzei (steps 4 and 5) (2012). The first author conducted all steps of the analysis and discussed interpretations with the second and third author, who had read the transcripts and field notes. The analysis was conducted in the following (simplified) steps: (1) Reading the transcripts and field notes to get an overall impression of the material and identifying preliminary themes (e.g., psychiatric firefighting, person-dependent care). (2) Extracting meaning units from the transcripts and the field notes and sorting them into codes (e.g., vulnerability, shame, loss of self-worth, uncertainty, feeling understood, not feeling understood). (3) Sorting the codes into three preliminary code groups: patient position, equality vs. lack of equality in patient-professional meetings, dignity in care. (4) We then used the theory of liminality (Turner, 1967; 1977/1991; Van Gennep, 1960/2004) in the interpretation of the patients’ experiences, and developed the final two code groups: vulnerable position as suicidal patient, recognizing vs. not recognizing the patient as a person. We then condensed the meaning within the code groups. (5) Finally, we summarized the content into meaningful descriptions and the two main findings; “Liminality and weakened sense of personhood,” and “Recognition of personhood.” We collaborated on developing the final descriptions, and we read transcripts and field notes during this hermeneutical process (moving back and forth between data and the literature) to ensure that the findings were grounded in the empirical data (Malterud, 2012, 2017).

### Ethical considerations

The Regional Committee for Medical and Health Research Ethics approved the study. The participants signed two informed consent forms, one for allowing the researcher to conduct participatory observation in the ward, and one for participating in an interview. Only two of the patients invited to participate declined the request. We only use the data collected in interactions with patients who agreed to participate and signed the consent form. JH informed the participants that they could withdraw from the study at any time prior to publication without giving a reason. The patients had the opportunity to contact her if they had any questions about the study or their participation. She had arranged with the patient’s therapist and/or nurse to be available in case the interview evoked mental distress and the patient needed a follow-up conversation. None of the patients needed follow-up after the interview. We treat the data in a confidential manner and present the information about the participants in such a way that they are not identifiable to others. We refer to the patients/women with fictitious names to protect their anonymity.

## Findings

We found that the patients’ experiences of suicidality and psychiatric hospitalization involve “Liminality and weakened sense of personhood,” and that “Recognition of personhood” is an important aspect of care. In this presentation, we use symbols to indicate when the findings are from interview (I), conversation (C), observation (O), or a combination of these data collection methods (I, C, O).

### Liminality and weakened sense of personhood

Our findings suggest that struggling with suicidality and being in the position as a patient in a psychiatric acute ward can be understood as being in a liminal phase and place with a weakened sense of personhood.

The women experienced suicidality to varying degrees while they were in the hospital (I, C). Some of them thought of suicide a lot and still wanted to die. Some patients experienced less suicidality for a while, for instance, because feeling well-taken care of. Then, it could increase, impulsively without any known reason to them, or, for example, because of feeling lonely and not cared for by the professionals (I, C). Several patients made plans or attempted to harm themselves, and two of them shared experiences of attempting to take their own lives (I, C). Greta felt that everything was black:

.. in the last weekend, and the days before that, it was really just all black. I felt like I was just here [in the acute ward], in a kind of waiting room, so that I could take my life at any point in time. (…) I had a couple of like impulsive attempts on Sunday. (…) But it, I wasn’t quite able to do it, so (I).

Being admitted to a psychiatric acute ward was thus not experienced as being in a safe place where she could be helped and prevented from taking her own life, but rather as being in a waiting room before her final suicide. Her description of being in a waiting room particularly illustrates a liminal experience—being in a between phase and place where she was vulnerable and uncertain whether she would survive. Everything was still black and being in the hospital had not yet led to a different and more positive mindset. She had little hope (I).

Several women shared similar experiences of hopelessness, feeling that everything was black, or that they had reached rock bottom (I, C). Some patients described loss of meaning, and a few seemed to lack the desire to live. Several of them felt unsuccessful, thinking they had failed in mastering life and staying well. The patients expressed uncertainty about their future, and some feared they would not become the person they were before their suffering started and that they would have a lower level of functioning in everyday life. Furthermore, some of the women made statements such as “I am not lovable,” or “I do not deserve anything,“ or “I am not good at feeling like a burden” (I, C). These experiences illustrate the patients’ negative self-perceptions. One of them, Filippa, felt ashamed because of the situation she was in, and her emotional distance to her children seemed particularly difficult:

.. become very ‘flat’ in relation to other people, and … but that it was going to happen with my kids, that, I never thought would happen (…) it has been painful to realize, that I do not miss them. I don’t quite understand it, how that is possible (…) very scary and … because then I have in a way become another person (…) it is quite awful (I).

The feeling of disconnection from her own children was new to her, it was scary and distressing and made her think that she had become another person. In a later conversation she had with her therapist, she wondered whether not missing her children could mean that she was an evil mother (C, O). She probably felt that she had failed in her role as a good and caring mother. The patients’ suffering and suicidality involved a weakened sense of personhood, with a changed sense of self and a significant loss of self-worth. The experiences illustrate liminality—being in a between phase, in a temporary “dark state and place” in life, where they experience despair, existential struggles and uncertainty related to who they are as persons, who they will become, what they mean to others, and what the future will bring, if they at all have a future (I, C).

Although most patients stated that they benefited from staying in the hospital when they felt treated well and cared for by the professionals, their experiences also indicate that the position as a patient in a psychiatric acute ward can pose some challenges that can be hard to deal with (I, C). Several women had difficulties in expressing their needs, and some of them were used to conceal their pain and to maintain a façade. All of them thought it was hard to contact the nurses when they needed care and support (I). At times, several of them stayed in their rooms and their feelings of loneliness and hopelessness increased (I, C, O). When Johanna was admitted to the ward, she felt completely stranded:

.. it was difficult for me here the two first days, because it was very much based on me asking for help and (…) Especially then, I wished that someone came and knocked [on the door]. (…) That, I remember very well that I wished for, that I felt completely stranded. Even though I was not, it was indeed people there. But I couldn’t go out myself and ask for help (I).

Staying in the room and not being able to seek help and support from the nurses increased her pain. She also said it made her feel even more like she had failed; approaching the nurses and ask for help was another thing she could not manage (I). Her experience of feeling stranded in the room illustrates a sense of liminality—being in a liminal (in between) phase and place where she felt isolated, helpless, and anxious—needing care, but not being able to seek it.

Further, several women said it was difficult to speak their minds, particularly if they disagreed with the professionals or were dissatisfied with treatment and care (I, C). Some of them had negative experiences from previous stays in the acute ward and feared conflicts with the therapist and nurses or other negative consequences (I, C). One patient, Iris, pointed to several restrictive conditions that could influence patients’ situation and make them more vulnerable:

.. you are locked in, you don’t decide by yourself whether you go out, and you don’t decide by yourself whether to be discharged, and they can define what they want within a frame where it is not visible to many others. And I have had experiences before that things went wrong then (…) if things go wrong, the communication gets so bad then, and then you are in it and have nothing to say, or nothing to do (I).

The therapists and nurses have a lot of power and authority, and the patients could feel powerless with limited autonomy (I, C). In addition, it seemed as if disagreements or conflicts could increase the patients’ sense of powerlessness. All the women were voluntarily admitted to the psychiatric acute ward, but several of them did not feel free to discharge themselves, and some could not leave the hospital when they wanted, presumably because of suicide risk (I, C). If the professionals think the patient’s life and health are endangered, they can refuse the patient to leave the ward based on the principle and act of necessity, penal legislation § 17. Going for walks outside or going to the store had to be arranged with the nurses, who needed time and opportunity to accompany them (I, C). Usually, the family could accompany the patients, but it seemed as if the nurses had different practices regarding it, as Dina illustrated:

.. sometimes I have been out with the family. But I was not allowed to do that yesterday. (…) Then I felt I got angry. I got so angry. In that sense, I think it is better to be, in a way, home, to know what I have. It is just about making the days go by, in a way, it is better that they go by fast. My goal is that I want to go home (I).

Being restricted evoked anger and frustration in her and made it difficult for her to be there. Dina and some of the other patients had several examples where they felt they had little influence on their own situation and course of treatment, which seemed to disempower them and made them feel small (I, C). Sometimes, sadness also appeared in their body language when they spoke of this (O). Thus, it seemed as if a weakened sense of personhood not only relates to their experiences of suicidality but also to their position as patients. Liminality is indicated in their temporary position as patients in a locked psychiatric ward, where they experienced a change of status with less authority over their own lives and ambivalence about treatment and care. They wanted to receive help, but at the same time, they wanted to escape such an uncomfortable and liminal phase and place, moving on to a hopefully better state and place in their life.

Summarized, these findings illustrate that patients’ experiences of suicidality and hospitalization in a psychiatric acute ward involve liminality and a weakened sense of personhood. That is, experiencing suicidality involved being in a temporary different and painful state where the individual may experience hopelessness, changed sense of self, loss of self-worth, loss of meaning, emotional disconnection from themselves and other people, ambivalence, and insecurity. Further, even though the patients also had experiences of feeling safe and cared for in the hospital, being admitted to a locked psychiatric acute ward meant being in a temporary different place where the patient had a different/lower status and experienced less authority, insecurity, discomfort, and anxiousness.

### Recognition of personhood

The findings suggest that recognition of personhood is an important aspect of care. The patients had both positive and negative experiences related to this finding. Thus, we describe it under the subheadings “experiencing recognition of personhood,” and “experiencing lack of recognition of personhood”.

#### Experiencing recognition of personhood

Experiencing recognition of personhood means that patients feel recognized, verbally and non-verbally, as an equal and valuable human being, and experience being taken seriously, respected, and understood. It also involves experiences of being supported and taken care of when needed (I, C).

The patients appreciated that the nurses recognized their suffering and cared for them when needed (I, C). Iris shared this example:

.. I had a really tough period (…) then one of the nurses came in and sat down on the floor and talked to me. (…) he *saw* that I was in pain and somehow he managed to get out of me what it was about. And then I was kind of allowed to talk a little about it. And then he led the conversation to something else, which was a little more cheerful. (…) he made me talk about something else, so that I … well, got my head onto a different track. I think that was very nice. (…) But also that he just was there, was calm and in a way sat there on my level, [he] was in it [the pain] together with me (…) and he stayed in it too, as long as I needed it then (I).

Iris felt recognized, and she could talk about her pain in her own way and at her own pace. In a sensitive and empathic way, the nurse was a companion in the pain, and when appropriate, the nurse led her to a different and more positive mindset (I). The patient seemed to feel uplifted by the nurse’s approach and way of being. As other patients expressed in the interviews, Iris appreciated that the nurse sat next to her, being on the same level physically, and at the same time connecting emotionally. Several patients said it was important that the therapists and nurses did not have a top-down attitude, or that they did not stand over them while talking if they themselves were seated (I), as Beth illustrated: “... that they [professionals] do not go and overpower people, but that they sit down at the level of the one in question. If the patient lies in the bed or sits in a chair, do not stand over them” (I). Thus, she valued being met as an equal. Further, it appeared that professionals promoted a sense of equality by having a respectful and not a top-down attitude, and by placing their body at the same level as the patient’s body (I).

Body language is of significance regarding the experience of being recognized. Several patients noted it was positive when the nurses came all the way into their room when they checked upon them, and not only stood in the doorway (I). Furthermore, the patients appreciated eye contact with the professional, a smile, a handshake, and a careful touch such as a pat on their shoulder (I). Being recognized as a person also meant that professionals approached them using their names and continued to use their name during the conversation. Dina described: “... those who have just put a hand on my shoulder, or .*.. *or use my name. It hits me in a way, it hits me a little deeper. It’s a bit good, but at the same time a bit like … like breathing almost, to start with (I).” When the professional gently touched her and used her name, she was emotionally affected. She felt it was good in a way, but at the same time, such a personal approach in this unfamiliar setting also seemed to surprise her or startle her a bit.

The patients’ experiences of good meetings with the therapists and the nurses in the ward seemed to have a lot to do with the professionals perceiving them and approaching them as human beings and not as diagnoses (I). Camilla explained: “Just being seen as a human being, really, and not being seen as a diagnosis, and not that they just see the act, because … just getting a little understanding and a little empathy, it can do so much (I).” To meet therapists and nurses who recognized her as a person, who made efforts to understand her pain, and not only make judgements based on her diagnosis and behaviour was significant. Iris pointed to the importance of professionals remembering that they and the patients have a lot in common: “... to be able to remember that … we are actually people like themselves. Without being completely like them, because we are not, none of us are. And to understand that we have the same needs (I).” Patients are in a different situation and position than the professionals, yet, they are all people with the same basic needs. Several of the women said they appreciated doing everyday activities together with nurses, such as going outside for walks, going to the store, or going home to collect mail or other personal things (I, C). Such activities were meaningful and helped remind them of who they were as persons outside the hospital.

#### Experiencing a lack of recognition of personhood

Experiencing a lack of recognition of personhood means that patients do not feel recognized as an equal and valuable human being, and they do not feel respected or understood. It also involves experiences of feeling treated as an object, not being sufficiently informed or involved in decision-making regarding treatment and care, and not feeling taken care of by the professionals (I, C, O).

Sometimes, the patients struggled to be heard (O). After one of the conversations with her therapist, Helen looked sad and she said, “He does not listen to me (C, O).” As also experienced by other patients, the therapist had set the agenda for the conversation, and Helen felt she had little influence on their meeting and on her course of treatment. She seemed discouraged and thought that treatment and care was more according to the system’s and the mental health workers’ terms than according to her needs (C, O). Helen experienced several challenging meetings with therapists and nurses where she did not feel recognized, respected, or understood (I, C). These experiences made her feel worse and contributed to self-harm, increased thoughts of suicide and even a suicide attempt (I). She had visible injuries (O). Getting enough time to recover was essential, but Helen learned this was difficult in this acute setting (I, C, O). Several patients pointed to the importance of getting enough time, and one of them, Camilla, had some very short-term hospitalizations which did not really help her: “... one does not get well by firefighting (I).” She needed more time and more help and felt that this kind of “psychiatric firefighting” was not sufficient and led to readmissions.

The women shared other examples of not being recognized, for instance when the nurses checked upon them at certain time intervals and just looked into their room without talking to them, as Anna illustrated: “...I often feel that I am just … I don’t know, inventory that they should check is here, in a way (I).” Lack of recognition made her feel like an object, as if she blended in among the furniture and was invisible as a person. In such situations, she felt as if the nurses did not care about her, which contributed to increased negative self-perceptions and thoughts of suicide. Once, she harmed herself in her room. She wished that the nurses had more time to be with her and care for her (I, C). Iris described another example where she felt treated like an object: “There is a feeling like not being seen and respected. To be treated as an object (…) the feeling that they don’t look at you as a human being, they are just handling you, in a way, as if they were putting together a jigsaw puzzle or hanging up a mirror (I).” She felt as if she was managed as a thing rather than being cared for as a person, which made her uncomfortable, angry, and a little dismissive of the professionals (I).

Several patients noted that care was person-dependent, i.e., depending on the individual therapist and nurse and his/her competence and/or attitude, and that they connected better with some professionals than others (I, C). Furthermore, there were some challenges related to the context and how the care was organized in the ward (as described in Research context), which made it difficult to establish good patient-professional relationships and maintaining continuity of care (I, C, O). Several patients commented on this. Dina reflected on why they [the management] had chosen to let the therapists and nurses rotate between three wards: “I don’t understand what … I don’t understand how they really intended it. Is it because, these are just thoughts I have had, is it because it can be very heavy in psychiatric, like acute [ward], that in order for it not to be too heavy for them, they have to rotate in a way? (I)” She wondered if caring for patients could be so emotionally straining that the professionals had to rotate between the wards to ease the burden. Such an organization did not seem as the most appropriate to meet her and other patients’ needs (I, C, O).

Summarized, these findings illustrate the importance of professionals recognizing the patients’ personhood verbally and nonverbally. Recognition of personhood contributed to strengthen the patient’s self-worth and to empower the person while being in a position with less authority and autonomy. Lack of recognition of personhood seemed to increase the patients’ suffering and hopelessness and disempowering the person. At worst, it could lead to increased self-harm and suicidality.

## Discussion

This study indicates that patients’ experiences of suicidality and hospitalization in a locked psychiatric acute ward involve liminality and weakened sense of personhood, and that an important aspect of care is to recognize and strengthen the patients’ personhood. To our knowledge, liminality is a concept not previously described in studies exploring patients’ experiences of suicidality and psychiatric hospitalization, and it adds a new and meaningful perspective to such experiences.

In this study, liminality illustrates some of the pain, vulnerability, and insecurity involved in suicidality and in the position as a patient in a locked psychiatric acute ward. Liminality can be understood as being in limbo, in a between phase and place, where the person is uncertain of the outcome (Barrett, 1998). Most of the patients in our study communicated uncertainty with regard to their future, whether they at all would recover and have a future. Some patients expressed existential struggles and uncertainty related to whom they were as persons, who they would become and what they meant for others. As psychiatric patients, they were temporarily separated from the outside world and had a changed status with less authority and autonomy, which could cause uncertainty, discomfort, and frustration. Two patients’ use of metaphors particularly illustrated the liminality in their situation. One metaphor illuminated how being in the ward was like being in a kind of waiting room before the final suicide. As if the patient was in a space between life and death, her life was on hold and she was vulnerable, waiting until her final act of suicide. Although it seemed as if she had decided to take her own life at some point, her use of “waiting room” also indicated ambivalence and uncertainty—perhaps something else than suicide could be the outcome when she left this liminal “in-between” space. The other metaphor showed how being alone in the room and feeling distressed and helpless was experienced as being completely stranded. As if the patient felt emotionally and physically stuck in the pain, trapped in her own room with nowhere to go and no one to turn to. The patients’ use of visual imagery evokes emotions and contributes to a deeper understanding of their difficulties. These metaphors as well as other descriptions of their experiences of being suicidal and hospitalized in a psychiatric acute ward indicate that there might be *degrees of liminality* (Thomassen, 2009, pg.18). The patients are in a liminal position when they are hospitalized, but there are also moments or periods when their suffering and suicidality are more intense, and then the experience of liminality (and of weakened personhood) is more evident too. Further, the patients’ experiences illustrate the intra-and interpersonal aspects involved in liminality and suicidality.

Our study suggests that even though the experience of suffering and suicidality is individual and unique for each patient, the development and maintenance of suicidality are very much relational and dialogical. Such aspects are not sufficiently emphasized in clinical practice. As this study shows, some of the patients’ suicidality were closely related to the interactions with the therapists and the nurses. That is, when the patients felt recognized and well-taken care of by the professionals their pain was relieved, whereas when the patient did not feel recognized and not well-taken care of by the professionals, their pain worsened. It could even lead to self-harm and increased suicidality. This is like what other researchers have found; lack of a good connection with the professional and lack of confirmation could lead to increased hopelessness and suicidality among patients (Berg et al, 2017; Samuelsson, Wiklander, Åsberg, & Saveman, 2000; Talseth et al., 1999). Several researchers emphasize that suicidality should be understood as an act of communication where it is part of an ongoing internal and external dialogue (Fleischer, 2000; Hustvedt, 2013; Knizek & Hjelmeland, 2007). Suicidality is thus not something located in the individual, but something that develops *between* people (Knizek & Hjelmeland, 2007). For this reason, establishing a dialogue with the suicidal person and exploring what meaning the suicidality has is crucial (Fleischer, 2000). Furthermore, some researchers suggest that suicidality is more common among persons who have adverse early attachment experiences and who have developed an insecure attachment style with subsequent interpersonal problems (Miniati, Callari, & Pini, 2017; Stepp et al., 2008). This vulnerability in relationships, as well as the suicidality itself (which may involve intense feelings of worthlessness and disconnection from self and others), indicates that suicidal patients, perhaps more than others, need to be confirmed and recognized as valuable persons.

Our study demonstrates the importance of recognition (recognition of personhood) in the care of patients struggling with suicidality. This finding is in keeping with previous research studying suicidal patients’ experiences (Berg et al. 2017; Hagen et al., 2018; Sellin et al., 2017; Vatne & Nåden, 2018), and other literature emphasizing the patient perspective in treatment and care (Cutcliffe & Barker, 2002; Cutcliffe & Stevenson, 2007; Jobes, 2006; Michel & Jobes, 2011). In a previous study, we interviewed former suicidal patients, and the participants emphasized the importance of a sense of companionship with the professional where they could feel safe enough to share their problems and suicidality (Hagen et al., 2018). Furthermore, the persons pointed to the importance of encountering therapists and nurses who treated them with respect, who recognized their needs and who made them feel like valuable fellow beings (Hagen et al., 2018). In the present study, the patients’ need for recognition and compassionate care was even more evident as they shared their experiences while being in an ongoing suicidal crisis and in a psychiatric hospital. They were vulnerable and experienced difficulties related to both their suicidality and being psychiatric patients. Our finding “liminality and weakened sense of personhood” illustrates some of the patients’ pain and struggles, and their safety and well-being depended on being cared for by therapists and nurses who understood them and responded to their particular needs when they needed. This study illustrates the importance of professionals’ verbal and nonverbal behaviour. The therapists’ and nurses’ way of being and how they acted physically (e.g., how nurses acted when they checked upon patients at certain time intervals) influenced how patients experienced the care.

Our findings are based on women’s experiences. In our research setting, most of the acute psychiatric patients struggling with suicidality were women, which was not surprising considering that more women than men engage in suicidal behaviour in our part of the world. We have reflected upon whether the participants’ gender may have influenced our findings. For example, although we consider the patients’ need for respect and recognition as fundamental for all people seeking help, and even though the patients in this study had experiences of good care, we were a bit puzzled and discouraged by some of these women’s struggles to be heard and recognized. Some of them seemed to hold back the strong emotions and frustration they sometimes felt in meetings with therapists and nurses, partly because of previous negative experiences. The women wished to establish connections with the professionals, they wanted the therapists and the nurses to listen to them and to care for them when they needed, and they wanted to collaborate, which reflect an emphasis on relational values (Jordan, 2004). However, the patients encounter professionals working within a mental health system that emphasizes standardization, individuality (including individual pathology), independence, and hierarchy, where the professional is positioned as the “expert” with most authority; thus, relational values appear undermined (Jordan, 2004). Furthermore, Gilligan (1982/1993, 1990), who studied women’s psychology, found that girls’ and women’s thinking and speaking reflected a focus on attachment and relationships, but during girls’ development into adulthood, they learn to suppress and silence their thoughts, emotions, and knowledge of reality and relationships so they conform to patriarchal structures and dominant social norms. Then, they face problems of relationship, because how can they adapt to the cultural structures and stay connected with others and at the same time stay connected with themselves and their own voices? (Gilligan, 1990). Although Gilligan developed this theory over 30 years ago, it may still apply to our society. The women’s experiences in this study raise issues that are important to explore further.

### Conclusions and clinical implications

Our study shows some significant difficulties that women struggling with suicidality experience during their stay in a psychiatric acute ward, and that therapists and nurses do not always provide the kind of care the patients need. Based on the perspectives of the patients in our study, some aspects of care should be improved. Professionals need to be more aware of and responsive to the pain and uncertainty involved in both the suicidal experience and to the position as a patient in a locked psychiatric ward. The therapists and nurses should be more aware of the importance of their way of being (including verbal and nonverbal behaviour), and how their care (or lack thereof) influences the patient and his/her suicidality. The professionals need to acknowledge more the relational aspect of suicidality and the importance of recognition of personhood in the care of the patients. The services would benefit from using more resources on education, training, and supervision of the professionals and ensuring sufficient staffing in the ward. In addition, the management ought to organize the mental health service in a way that promotes good patient-professional relationships and maintain continuity in care.

### Strengths and limitations of the study

The study provided both observational and interview data (though mostly based on interviews and conversations), and JH met most of the patients several times, while they still experienced suicidality and were in a psychiatric hospital. JH has clinical experience as a mental health nurse, and all authors have research experience on this topic. This experience is a strength, but in keeping with Finlay (2008), we had to be aware of our preunderstandings and reflect upon them to prevent them from blocking for new understandings based on the participants’ unique experiences. The findings provide valuable insights into the experiences of women admitted to the psychiatric acute ward. JH had to recruit the participants through the professionals, which meant that some patients were not informed about the study (e.g., the nurse/therapist forgot to inform them before they were discharged, or they seemed reluctant to inform a few patients, probably because they assumed it could be stressful for them to participate). The findings are closely connected to the context in which they were developed but may apply to other similar settings and to other persons experiencing suicidality and psychiatric hospitalization. Thus, to some extent, the findings can be transferable (Malterud, 2017; Polit & Beck, 2010). However, the assessment of transferability largely depends on the value that readers assign to the research (Polit & Beck, 2010).

### Future research

We suggest that future studies explore the experiences of suicidal patients (men and women) over a longer period where they can be interviewed several times, including after discharge from the hospital. Relational aspects of care, especially regarding the patients’ challenges of being heard and recognized need to be explored further. Researchers could further study the concept of liminality in relation to individuals’ experiences of suicidality in different contexts, providing more insight into the suffering and uncertainty, the sense of being in a “betwixt and between” phase and place in life, and also study the potential transformation involved in such a process. Exploring the patients’ use of metaphors would be particularly interesting in that respect.

## References

[cit0001] Barrett, R. J. (1998). The ‘schizophrenic’ and the liminal persona in modern society. *Culture, Medicine and Psychiatry*, 22, 465–10. 10.1023/a:1005393632053 10063468

[cit0002] Berg, S. H., Rørtveit, K., & Aase, K. (2017). Suicidal patients’ experiences regarding their safety during psychiatric in-patient care: A systematic review of qualitative studies. *BMC Health Services Research*, 17, 73. 28114936 10.1186/s12913-017-2023-8 PMC5259991

[cit0003] Berglund, S., Åström, S., & Lindgren, B. M. (2016). Patients’ experiences after attempted suicide: A literature review. *Issues in Mental Health Nursing*, 00, 1–12. 10.1080/01612840.2016.1192706 27327200

[cit0004] Biong, S., & Ravndal, E. (2009). Living in a maze: Health, well-being and coping in young non-western men in Scandinavia experiencing substance abuse and suicidal behavior. *International Journal of Qualitative Studies on Health and Well-being*, 4, 4–16.

[cit0005] Bruce, A., Sheilds, L., Molzahn, A., Beuthin, R., Schick-Makaroff, K., & Shermak, S. (2014). Stories of liminality. Living with life-threatening illness. *Journal of Holistic Nursing*, 32(1), 35–43. 23926216 10.1177/0898010113498823

[cit0006] Cutcliffe, J. R., & Barker, P. (2002). Considering the care of the suicidal client and the case for ‘engagement and hope’ or ‘observations’. *Journal of Psychiatric and Mental Health Nursing*, 9, 611–621. 12358715 10.1046/j.1365-2850.2002.00515.x

[cit0007] Cutcliffe, J. R., & Stevenson, C. (2007). *The care of the suicidal person*. Philadelphia: Churchill Livingstone Elsevier.

[cit0008] Digby, R., Lee, S., & Williams, A. (2017). The liminality of the patient with dementia in hospital. *Journal of Clinical Nursing*, 27, e70–e79. 28493647 10.1111/jocn.13869

[cit0009] Finlay, L. (2008). A dance between the reduction and reflexivity: Explicating the “phenomenological psychological attitude”. *Journal of Phenomenological Psychology*, 39(1), 1–32.

[cit0010] Fleischer, E. (2000). *Den talende tavshet: Selvmord og selvmordsforsøg som talehandling [the speaking silence: suicide and suicide attempt as a speech act]*. Odense: Odense Universitetsforlag.

[cit0011] Gibbons, S. W., Ross, A., & Bevans, M. (2014). Liminality as a conceptual frame for understanding the family caregiving rite of passage: An integrative review. *Research in Nursing and Health*, 37(5), 423–436. 25176315 10.1002/nur.21622 PMC4180249

[cit0012] Gilligan, C. (1990). Joining the resistance: Psychology, politics, girls, and women. *Michigan Quarterly Review*, 29, 501–536.

[cit0013] Gilligan, J. (1982/1993). *In a different voice. Psychological theory and women’s development*. Cambridge: Harvard University Press.

[cit0014] Hagen, J. (2018). Care and control-Exploring experiences and perceptions of treatment and care of suicidal inpatients in psychiatric wards. Doctoral thesis. Norwegian University of Science and Technology, Faculty of Medicine and Health Sciences, Department of Mental Health. ISBN 978-82-326-2893-3

[cit0015] Hagen, J., Knizek, B. L., & Hjelmeland, H. (2018). Former suicidal inpatients’ experiences of treatment and care in psychiatric wards in Norway. *International journal of Qualitative Studies on Health and Well-being*, 13, 1461514. 29652227 10.1080/17482631.2018.1461514 PMC5906934

[cit0016] Hjelmeland, H., & Knizek, B. L. (2017). Suicide and mental disorders: A discourse of politics, power, and vested interests. *Death Studies*, 41(8), 481–492. 28535129 10.1080/07481187.2017.1332905

[cit0017] Hustvedt, S. (2013). Suicide and the drama of self-consciousness. *Suicidology Online*, 4, 105–113.

[cit0018] Jackson, A. Y., & Mazzei, L. A. (2012). *Thinking with theory in qualitative research: Viewing data across multiple perspectives*. New York: Routledge.

[cit0019] Jobes, D. A. (2006). *Managing suicidal risk. A collaborative approach*. New York: The Guilford Press.

[cit0020] Jordan, J. V. (2004). Toward competence and connection. In J. V. Jordan, M. Walker, & L. M. Hartling (Eds.), *The complexity of connection. Writings from the Stone Center’s Jean Baker Miller Training Institute* (pp. 11–28). New York: The Guilford Press.

[cit0021] Knizek, B. L., & Hjelmeland, H. (2007). A theoretical model for interpreting suicidal behaviour as communication. *Theory & Psychology*, 17(5), 697–720.

[cit0022] Kvale, S., & Brinkmann, S. (2009). *Interviews: Learning the craft of qualitative research interviewing* (2nd ed.). Thousand Oaks: Sage Publications.

[cit0023] Malterud, K. (2012). Systematic text condensation: A strategy for qualitative analysis. *Scandinavian Journal of Public Health*, 40, 795–805. 23221918 10.1177/1403494812465030

[cit0024] Malterud, K. (2017). *Kvalitative forskningsmetoder for medisin og helsefag* [Qualitative research methods for medicine and health] (4th ed.). Oslo: Universitetsforlaget.

[cit0025] Marsh, I. (2010). *Suicide: Foucault, history and truth*. Cambridge: Cambridge University Press.

[cit0026] Marsh, I. (2016). Critiquing Contemporary Suicidology. In J. White, I. Marsh, M. J. Kral, & J. Morris (Eds.), *Critical suicidology: Transforming suicide research and prevention for the 21st century* (pp. 15–30). Vancouver: UBC Press.

[cit0027] Michel, K., & Jobes, D. A. (2011). *Building a therapeutic alliance with the suicidal person*. Washington DC: American Psychological Association.

[cit0028] Miniati, M., Callari, A., & Pini, S. (2017). Adult attachment style and suicidality. *Psychiatria Danubina*, 29(3), 250–259. 28949306 10.24869/psyd.2017.250

[cit0029] Norwegian Directorate of Health and Social Affairs. (2008). *Nasjonale retningslinjer for forebygging av selvmord i psykisk helsevern* [National guidelines for the prevention of suicide in mental health care]. IS-1511. Oslo: Author.

[cit0030] Norwegian Institute of Public Health. (2019). *Dødsårsaksregisteret-statistikk. Selvmord etter kjønn, alder og dødsmåte* [Cause of death registry-statistics, suicide by gender, age and way of death]. [uploaded 6.9.2019]. Oslo: Author.

[cit0031] Polit, D. F., & Beck, C. T. (2010). Generalization in quantitative a d qualitative research: Myths and strategies. *International Journal of Nursing Studies*, 47, 1451–1458. 20598692 10.1016/j.ijnurstu.2010.06.004

[cit0032] Samuelsson, M., Wiklander, M., Åsberg, M., & Saveman, B. I. (2000). Psychiatric care as seen by the attempted suicide patient. *Journal of Advanced Nursing*, 32, 635–643. 11012806 10.1046/j.1365-2648.2000.01522.x

[cit0033] Sellin, L., Asp, M., Wallsten, T., & Wiklund-Gustin, L. (2017). Reconnecting with oneself while struggling between life and death: The phenomenon of recovery as experienced by persons at risk of suicide. *International Journal of Mental Health Nursing*, 26, 200–207. 27417106 10.1111/inm.12249

[cit0034] Stepp, S. D., Morse, J. Q., Yaggi, K. E., Reynolds, S. K., Reed, I., & Pilkonis, P. A. (2008). The role of attachment styles and interpersonal problems in suicide-related behaviors. *Suicide and Life-Threatening Behavior*, 38(5), 592–607. 19014310 10.1521/suli.2008.38.5.592 PMC2823257

[cit0035] Talseth, A. G., Jacobsson, L., & Norberg, A. (2001). The meaning of suicidal psychiatric inpatients’ experiences of being treated by physicians. *Journal of Advanced Nursing*, 34, 96–106. 11430612 10.1046/j.1365-2648.2001.3411729.x

[cit0036] Talseth, A. G., Lindseth, A., Jacobsson, L., & Norberg, A. (1999). The meaning of suicidal psychiatric inpatients’ experiences of being cared for by mental health nurses. *Journal of Advanced Nursing*, 29, 1034–1041. 10320485 10.1046/j.1365-2648.1999.00990.x

[cit0037] Thomassen, B. (2009). The uses and meanings of liminality. *International Political Anthropology*, 2(1), 5–27.

[cit0038] Turner, V. W. (1967). Betwixt and between: The liminal period in rites de passage. In *The forest of symbols: Aspects of Ndembu ritual* (pp. 93–111). Ithaca (NY): Cornell University Press.

[cit0039] Turner, V. W. (1977/1991). *The ritual process. Structure and anti-structure*. Ithaca: Cornell University Press.

[cit0040] Van Gennep, A. (1960/2004). *The rites of passage*. New York: Routledge.

[cit0041] Vatne, M., & Nåden, D. (2018). Experiences that inspire hope: Perspectives of suicidal patients. *Nursing Ethics*, *25*(4), 444–457. 10.1177/0969733016658794 27521246

[cit0042] Walby, F. A., Myhre, M. Ø., & Kildahl, A. T. (2018). *1910 døde pasienter: Selvmord i psykisk helsevern og tverrfaglig spesialisert rusbehandling 2008–2015 – En nasjonal registerstudie* [1910 dead patients: Suicide in mental health and substance misuse services 2008–2015 – A national registry study]. (Report). Oslo: The Norwegian Surveillance System for Suicide in Mental Health and Substance Misuse Services.

[cit0043] White, J., & Morris, J. (2019). Re-thinking ethics and politics in suicide prevention: Bringing narrative ideas into dialogue with critical suicide studies. *International Journal of Environmental Research and Public Health*, 16, 3236. 10.3390/ijerph16183236 PMC6766026 31487801

